# A Comparison of Implicit and Explicit Motor Sequence Learning in Patients with Relapsing-Remitting Multiple Sclerosis

**DOI:** 10.3390/sports5020034

**Published:** 2017-06-07

**Authors:** Maliheh Sarabandi

**Affiliations:** Department of Physical Training, Faculty of Human Sciences, University of Zabol, Zabol 009854, Iran; maliheh.sarabandi@gmail.com; Tel.: +98-93-8507-4737

**Keywords:** multiple sclerosis, implicit learning, explicit learning

## Abstract

This study tends to assess implicit and explicit types of motor learning in patients with Multiple Sclerosis (MS) and normal peers by means of a serial reaction time. Sample size was 15 for each group and Samples included 30 patients with MS and 30 normal peers and were assigned to implicit and explicit learning groups. A repeated measures ANOVA was used for measuring reaction time and response error, and a paired samples *t*-test was used to compare regular and irregular sequence data in each group. Comparison of these two types of learning in speed (response time) and accuracy (number of errors) showed the number of errors (*P* = 0.012) and response time (*P* = 0.012) in the implicit motor learning group of MS patients and the number of errors (*P* = 0.096) and response time (*P* = 0.954) in the explicit motor learning group of MS patients. Moreover, comparison showed the number of errors (*P* = 0.008) and response time (*P* = 0.05) in the implicit group of normal peers and the number of errors (P = 0.011) and response time (*P* = 0.442) in the explicit group of normal peers. The results showed that explaining and describing the task is less effective at training the motor sequence of MS patients and that these patients benefit more from implicit learning.

## 1. Introduction

Multiple sclerosis (MS) is a chronic, progressive, disabling disorder characterized by immune-mediated demyelination, inflammation, and neurodegenerative tissue damage in the central nervous system (CNS), associated with frequent exacerbations and remissions of neurologic symptoms and eventual permanent neurologic disability. The vast majority of patients suffer from recurrent relapses followed by remissions of neurological symptoms, especially in the early course of disease [1]. People learn with certain rules in their environment, without being aware of this learning. The acquisition of sequence of information and events, regardless of learning or awareness of what has been learned, represents implicit learning [2]. In this type of learning, people gain skill in a task without explicit knowledge of that task [3]. Explicit knowledge refers to explanations and instructions on how to perform a motor task, which represents another type of learning that is explicit learning [4]. In explicit learning, the learner is clearly informed of the purpose of learning, and they think about learning processes. This process necessarily consumes a large part of cognitive resources [5]. The ability to learn and produce sequential motor actions, such as typing, riding a bike, and learning how to operate new electronic wheelchair, is a remarkable human ability. However, this ability may be damaged in patients with brain injuries or degenerative diseases such as MS. Multiple sclerosis is a common disease of the central nervous system characterized by widespread presence of lesions affecting the brain, the spinal cord, and optic nerves [6]. In this disease, myelin, which is involved in transmitting nerve impulses along nerve fibers, is damaged. Nerve impulses will be transmitted with less disruption if the damage is minor; otherwise, scar-like tissue replaces myelin and hence the transmission of nerve impulses may be completely disrupted. This disease is called multiple sclerosis because it causes lesions in large areas of the nervous system [7], and it is associated with a wide range of visual, sensorymotor, cognitive, and neurological disorders [8,9]. RRMS, the most common disease course, is characterized by clearly defined attacks of new or increasing neurologic symptoms. These attacks—also called relapses or exacerbations—are followed by periods of partial or complete recovery (remissions). During remissions, all symptoms may disappear, or some symptoms may continue and become permanent. However, there is no apparent progression of the disease during periods of remission. At different points in time, RRMS can be further characterized as either active (with relapses and/or evidence of new MRI activity) or not active, as well as worsening (a confirmed increase in disability over a specified period of time following a relapse) or not worsening. Relapsing-remitting MS is defined by inflammatory attacks on myelin (the layers of insulating membranes surrounding nerve fibers in the central nervous system (CNS)), as well as the nerve fibers themselves. During these inflammatory attacks, activated immune cells cause small, localized areas of damage which produce the symptoms of MS. Because the location of the damage is so variable, no two people have exactly the same symptoms [10].Based on neuropsychological tests, approximately 43%–70% of MS patients suffer from cognitive disorders [11]. Cognitive disorders are the most common clinical characteristics of MS patients observed in initial to most severe cases of the disease and significantly affect functional status of the patients [11,12]. Rao et al. [13] showed that MS patients who had cognitive problems participated less in social and professional activities, had difficulty doing housework, and were more vulnerable to mental diseases compared to those with physical disabilities. In these patients, cognitive disorder is associated with certain impairments in learning and memory, attention, information processing speed, executive functions, and visual-spatial functions [11,14,15]. Although motor sequence learning is essential for normal human motor functions, few studies have measured this skill in MS patients. In studies on neurological diseases such as Parkinson’s disease and Alzheimer’s disease, Wilkinson, Khan, and Jahanshahi [16] stated that both implicit and explicit learning is impaired in Parkinson’s disease. Machado-Vieira et al. [17] showed that explicit memory is severely destructed in Alzheimer’s disease, while implicit memory of events is maintained, and it can compensate impaired explicit memory through compensatory strategies. Gong et al. [18] suggested that patients with mild cognitive impairment exhibit impaired explicit memory performance. In particular, non-specific learning is destructed in MS patients with severe neurological disorder, while it is relatively preserved in patients with milder disorder [19,20,21]. Tacchino et al. [22] showed that MS patients with minimal disability exhibit fine motor impairment, and the ability to learn simple motor tasks short-term is almost preserved, while they exhibit problems in both explicit and implicit learning with higher and more prominent impairment in terms of implicit learning in more complex and specific tasks. Rao et al. [23] observed that implicit motor sequence learning was intact and preserved in patients compared to healthy controls. Rao [24] showed that these patients exhibit slow mental processing and memory. Lazeron et al. [25] concluded that processing speed in all cognitive areas, particularly memory, is impaired in these patients. Solari et al. [26] showed that the potential for learning motor skills is preserved, even in disabled patients. In this regard, Bonzano et al. [27] showed that people with MS could reduce response time through explicit learning in a repeated sequence. Focusing on explicit sequence learning in relapsing-remitting MS patients, Zahiri et al. [28] showed that explicit motor learning occurs less in this group compared to healthy peers. Bonzano et al. [27] showed that MS patients with minimal disabilities have impaired explicit motor-visual sequence learning compared to healthy peers; they also showed that explicit learning performance is significantly lower than that in the control group. Studying conscious and implicit information remembering, Scarrabelotti and Carroll [29] found no significant difference in a conventional implicit task between MS and control groups; however, the performance of the MS group was significantly lower under explicit instructions. Although learning disorders cause many problems for the individual and society, the exact nature of effective processes on these disorders is not well understood. Cognitive rehabilitation therapy, which emphasizes the restoration of lost capabilities or the improvement of intact compensatory functions, is a promising solution to eliminate the effects of these disorders in MS patients. One of the most useful exercises in sequential learning in various empirical studies is the serial reaction time task (SRTT). In this application, a square appears in 4 points, each time in each of the 4 corners of the screen, which can change into 4 colors (yellow, green, blue, and red). Each of these colors is labeled on the keyboard (P for blue, Q for yellow, Z for green, and M for red). The next square appears by pressing the relevant key. If one mistakenly presses key of other colors, the next square will appear again [4]. In this application, each sequence involves the iteration of seven squares totaling 70 squares, called a block, on which calculations and analyses are done. Seven blocks were used in this experiment. Iteration of colorful squares had two different patterns in each sequence. Squares in each sequence appeared with a random pattern that was determined by the application, and no logical relationship existed in order of appearance. The other pattern was regular in which blue, yellow, blue, red, yellow, green, and yellow appeared, respectively. This study considered reduced error or increased correct responses to stimuli as accuracy and response time as the speed of motor learning. The task given to subjects included 7 steps; Steps 2 and 6 were irregular, and other steps were regular and sequential. The time of each step (in thousandth of milliseconds) and the number of wrong responses to target stimuli were measured by the application. Response time was considered as a measure of learning speed, and the number of wrong answers was considered as a measure of learning accuracy. Increased response time considering random sequence implies understanding of a certain sequence of knowledge and information [30]. Sequences can be learned by appearance of stimuli, that is visual-spatial representation, or order of movements such as the order of pressing keys [31]. In fact, this task (SRTT) is used to identify a wide range of behaviors including cognitive and biological principles of learning and memory [3]. However, it is essential to understand lost and preserved capabilities of these patients to increase the effectiveness of cognitive rehabilitation therapy. Moreover, motor rehabilitation programs can directly benefit results of cognitive studies to restore physical function. Cognitive rehabilitations tend to reduce cognitive problems, improve the disease and promote positive biological changes in these patients [12]. Using the SRTT test, this study compares implicit and explicit motor sequence learning in both MS patients and healthy controls, and it is found that learning categories may be impaired in these patients. This study can provide information about these patients’ ability to learn motor skills and note the importance and necessity of implicit or explicit learning in rehabilitation situations.

## 2. Materials and Methods

This quasi-experimental study was based on convenient sampling. By measuring the standard deviation of previous studies [28], the sample size was set at 15 for each group (the explicit motor sequence learning group and the implicit motor sequence learning group); the samples were recruited from clients of treatment centers for special diseases. Moreover, 30 normal peers were assigned to two control groups of implicit and explicit learning. All patients and normal participants were randomly assigned to two groups of implicit and explicit learning. Table 1 and Table 2 list demographic data including age, course, and severity of the disease related to subjects and controls. All patients suffered from RRMS.

Average functional disability was calculated based on the extent of disability status scale (EDSS). The Expanded Disability Status Scale (EDSS) is a method of quantifying disability in multiple sclerosis and monitoring changes in the level of disability over time. It is widely used in clinical trials and in the assessment of people with MS. The scale was developed by a neurologist called John Kurtzke in 1983 as an advance from his previous 10 step Disability Status Scale (DSS). The EDSS scale ranges from 0 to 10 in 0.5 unit increments that represent higher levels of disability. Scoring is based on an examination by a neurologist [32]. In assessing this factor in this study, no significant difference between patients groups was not seen (0.07).

Moreover, participants were compared to 30 controls in terms of age, gender, Wechsler Intelligence Scale, and the MMSE test, and no significant difference was found among these groups. Using parallel group pilot study plan, 15 patients and 15 controls practiced implicit learning, and 15 patients and 15 controls practiced explicit learning. Inclusion criteria included RRMS diagnosed by a neurologist, minimum literacy (able to read and write), and right hand dominance. Exclusion criteria included severe memory impairment (score less than 21 on MMSE test), acute disease, and neurological diseases such as dementia or Alzheimer’s disease, cerebellar problems, and severe depression, and severe visual and hearing problems. None of the patients experienced motor problems during SRT exercises. All patients and control groups stated their consent to participate in the study by signing a consent form.

### 2.1. The Mini Mental State Examination (MMSE)

This examination was designed by Folstein (1975) [33]. This test is used for early evaluation of the cognitive status and has 11 items in two parts. The first part involves oral responding to orientation, memory, and attention questions. The second part requires reading and writing and involves the ability to name, following oral or written instructions, writing a sentence, and copying a shape. All questions were asked sequentially, and the total score is obtained from the sum of scores of the tasks carried out successfully. The maximum score was 30 and the cut point score was set to 23–24. A score between 18 and 23 indicates mild cognitive disorder, and a score below 17 indicates severe cognitive dysfunction [34]. Tierney et al. (1997) estimated correlation coefficient of sub-scales of the MMSE and the respected neuro-psychologic scales to be 0.5 and 0.6 [35].

### 2.2. Wechsler Adult Intelligence Scale

This scale is a set of memory tests for adults that was designed and standardized by Wechsler in 1945. The Wechsler Intelligence Scale has two forms, A and B, each including 7 sections and different themes (general and personal information, orientation, mind control, logical memory, digits repetition, visual memory, and learning associations). This scale was standardized by Sarrami (1993) in Iran with Cronbach’s alpha coefficient of 0.85 [36]. In the present study, to determinethe general status of the patients’ and control groups’ memory, Form A of the Wechsler memory scale was used. A Cronbach’s alpha of 0.76 was obtained in this study, and no significant difference was observed between patients and control groups (*P =* 0.06).

### 2.3. Subsection

#### Serial Reaction Time Task (SRTT)

**Figure 1:**
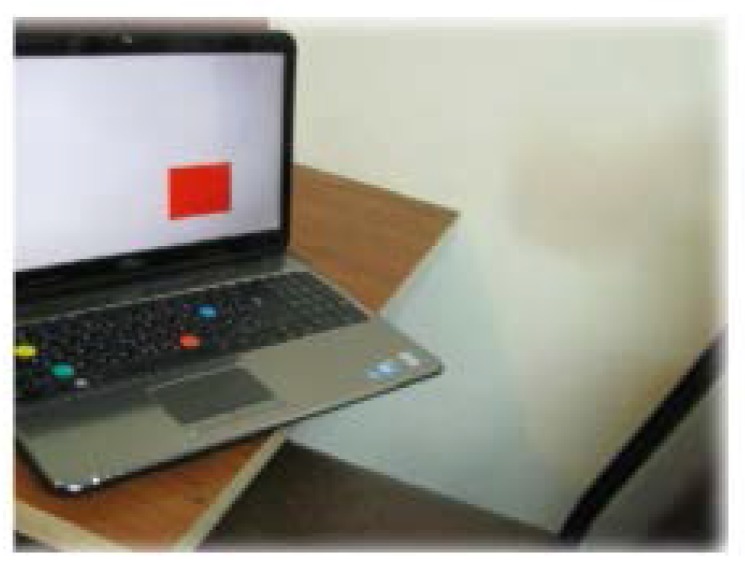
Test guideline.

The Persian version of the SRTT application was used in this study. Studies showed that this test is not dependent on culture in terms of reliability and validity. Moreover, data is recorded by a computer; thus, human error is not involved in recording [37]. Stimuli appear in the form of colored squares (yellow, green, blue, and red) on the screen; the subject needs to respond to stimuli by pressing the same color key by index finger of the right hand (dominant). Four keys are installed on the keyboard to respond to colors (P for blue, Q for yellow, Z for green, and M for red); the keys were labeled with the relevant colors (Figure 1). In this application, each sequence involves iteration of seven squares totaling 70 squares called a block on which calculations and analyses are done. Seven blocks were used in this experiment. Iteration of colorful squares had two different patterns in each sequence. Squares existing in each sequence appeared with a random pattern that was determined by the application and no logical relationship existed in order of appearances. The other pattern was regular in which blue, yellow, blue, red, yellow, green, and yellow appeared, respectively. This study considered reduced error or increased correct responses to stimuli as accuracy and response time as speed of motor learning. The task given to subjects included 7 steps; Steps 2 and 6 were irregular, and other steps were regular and sequential. The time of each step (in thousandth of milliseconds) and the number of wrong responses to target stimuli were measured by the application. Response time was considered as a measure of learning speed, and the number of wrong answers was considered as a measure of learning accuracy.

At this stage, the test involved 7 blocks. Block 3, 2, and 6 had a random arrangement and Blocks 5, 7, and 1 had a regular pattern. Here, they were not given any explanation of how the squares were repeating and were asked only that they see every square in the shortest possible time and press the corresponding key. Between every two sequential blocks, a1 min rest was given, and at the end, assign on the computer screen appeared indicating the end of the experiment. In the end, people were asked whether there was a repetitive pattern of squares or not. If there was a repetitive pattern, the people were asked to express it. If people, Correctly Expressed it, they were excluded from the implicit group and were included in the explicit group. Process in explicit group was similar to implicit motor learning group with the exception that pattern repeat squares and sequence and blocks was explained to the people. That is, in the first block, the sequence of appearance of colors were painted on the side, and then was removed.

## 3. Results

A paired samples *t*-test and a repeated measures ANOVA were used to analyze data obtained from SPSS in the four groups. The results are presented below.

### 3.1. Explicit Learning in MS Patients

#### 3.1.1. Reduced error

Based on the results obtained from the repeated measures ANOVA in explicit learning MS patients, there was no significant difference among the number of errors in regular steps (*P* = 0.094), and the paired *t*-test was not significant in irregular sequences (*P* = 0.838), which suggests that MS patients have similar errors in the explicit learning of irregular sequences.

#### 3.1.2. Reduced response time

There was no significant difference in response times of regular steps in explicit sequences for MS patients (*P* = 0.954). Moreover, the paired *t*-test was not significant for the response time of irregular sequences (*P* = 0.216).

### 3.2. Implicit Learning in MS Patients

#### 3.2.1. Reduced error

Based on results obtained from the repeated measures ANOVA, there was a significant difference among the number of errors in regular steps (*P* = 0.012); moreover, comparison of irregular steps by using the paired *t*-test showed no significant difference.

#### 3.2.2. Reduced response time

There was a significant difference in response times of regular steps in implicit sequence for this group (*P* = 0.012). Moreover, the paired *t*-test was not significant for response time of irregular sequences (*P* = 0.147).

### 3.3. Comparison of Explicit and Implicit Motor Sequence Learning

The main effect of the blocks showed a significant difference in learning accuracy (*P* = 0.006), while the main effect of the learning type showed no significant difference in accuracy (*P* = 0.844). Moreover, the main effect of the blocks showed a significant difference in learning speed (*P* = 0.009), while the main effect of the learning type showed no significant difference in speed (*P* = 0.431). A comparison of irregular steps (Blocks 2 and 6) showed no significant difference in accuracy (*P* = 0.512) and speed (*P* = 0.094) (Table 3 and Table 4).

## 4. Discussion and Conclusions

Implicit sequence learning involves learning a sequence of events, regardless of learning or awareness of what has been learned. This type of learning leads to many events and behaviors, including learning of new languages, understanding and production of complex sentences, walking with mobility aids, as well as various types of rehabilitation. In contrast, explicit learning includes conscious information of experiences. This study aimed at assessing the difference between implicit motor learning and explicit motor learning in patients with multiple sclerosis. Findings showed that implicit motor learning occurred in patients with MS, and there was no significant difference with normal peers. Motor performance of this group did not improve in regular blocks by exercise and showed a significant difference with the normal peer group. Explicit learning did not occur in the MS group, which probably indicates impaired learning in this group of patients. However, this type of learning occurred in healthy controls. Although no significant difference was found between the two groups, this learning was probably impaired. Motor performance of this group did not improve in irregular blocks by exercise and showed a significant difference with normal peers. Brain regions involved in learning sequential movements include cortex pre-motor, dorsal prefrontal cortex, and anterior supplementary motor area [38]. Nervous neuroanatomy in explicit learning is influenced by several subsets including activation of the cerebellum, thalamus, brain stem, and bilateral cerebellar vermin (related to eye movements). Linguistic and visual areas are activated in this type of learning, which reflects conscious strategies used in this type of learning [39]. Moreover, MS is characterized by extensive presence of lesions affecting brain, spinal cord, and optic nerves [12]; thus, it can cause explicit learning impairment in these patients. Due to the extensive nature of MS lesions in the central nervous system, this disease falls within a wide range of visual, sensory-motor, cognitive, and neurological symptoms [9,10]. Bonzanoet al. [27] showed that explicit learning of motor sequences is significantly reduced in these patients compared with healthy subjects. SRTT is a task that needs attention to stimulate quick response during the test. There is consensus that attention is impaired in these patients [40]. A team of researchers used tests that are influenced by motor impairment such as hearing test PASRT evaluating information processing speed and attention to measure response time. Based on the results, MS patients had weaker performance than controls in this task and their response time was slower. They considered this impairment to be caused by active memory impairment [41]. Random blocks in motor sequence learning are blocks of which people are not aware; thus, increased time in these blocks, in turn, reflects correct process of learning in regular blocks. On the other hand, relative time increase in these blocks indicates that only motor factors do not prolong response time, and factors related to learning play an important role in this regard. Studies show that MS patients are capable of motor learning [21,27]. The impairment observed in explicit learning is because of slow information processing and impaired active memory capacity, which is troubled when higher demand is imposed [27]. In explicit learning, conscious information requiring more attention and simultaneous mental processing for motor tasks increases demands on active memory; since the capacity of this memory is impaired in patients with MS, processes are not well protected [42]. Deroost et al. [43] showed that MS patients suffer major impairment in learning sequential motor skills; they claimed that sequential learning is impaired in patients with progressive MS under explicit learning. Patients, particularly patients with mild MS, were less successful in the awareness test in the explicit recovery of sequential items. In general, these results are consistent with previous studies in which it is claimed that implicit learning was intact [23] and that explicit learning was impaired in MS [28]. Among clinical representations of this disease, cognitive symptoms are clearer than other disabilities associated with this disease [44]. In explicit learning, the learner is clearly informed of the purpose of learning and thinks about the learning processes. This process necessarily consumes a large part of cognitive resources [5]; this can be a strong reason for the impairment of explicit learning processes in these patients. Cognitive dysfunctions are extensive in patients with MS and involve motor, cognitive, linguistic, learning, and executive functions [45]. Iaffaldanoet al. [46] concluded that cognitive impairment is the most well-known MS feature, even in early stages of the disease, and areas that are affected the most include sustained attention, information processing speed, and verbal memory. Because explicit learning training is not always suitable for certain groups of people, such as neurological patients, implicit learning can be a useful learning method in rehabilitation situations. The results obtained in this regard can open new windows as to how to train such patients and how they can learn to improve their learning processes. To sum up, the findings of this study, as shown by the different time measures, presented evidence of implicit learning of MS patients in the SRTT. Error and response time measures showed that MS patients were implicitly aware of repeated sequences. Considering different aspects that underlie human ability to dominate sequential motor functions, more information can be obtained about the impairment of processes in patients. The implicit learning of motor sequences is related to motor functions. People learn many sequential activities over time; however, they cannot explain how they learn these skills. In rehabilitation activities, for example, in learning to walk with mobility aids and other types of rehabilitation, implicit learning processes can be used for these patients. This type of learning, which is more sustainable than explicit learning, can be used in neurological patients, because its efficiency has been proven. In general, according to the results of the present research, it seems that explicit motor sequence learning is not impaired in MS patients.

## Figures and Tables

**Table 1:** Demographic variables of participants.

Variable/Group	MS Patient	Healthy Control	Test	*P*-Value
Gender	15 men and 15 women	15 men and 15 women	X^2^ = 0.40	0.309
Age	49.5 (7.9)	42.5 (7.2)	T(60) = 0.82	0.231
Wechsler Intelligence Scale	91.34 (5.23)	98.12 (3.87)	T(60) = 0.34	0.667
MMSE	26.8 (1.6)	27.5 (1.8)	T(60) = 0.43	0.491

**Table 2:** Clinical variables in explicit and implicit learning groups of MS patients.

Variable/Group	MS Patients (Implicit Learning) M(SE)	MS Patients (Explicit Learning) M(SE)	Test	*P*-Value
Disease duration (Years)	4.91 (1.05)	5.67 (2.21)	T(30) = 0.91	0.682
EDSS	5.8 (1.12)	5.3 (1.28)	T(30) = 0.28	0.781

**Table 3:** Results of the paired *t*-test and the repeated measures ANOVA in irregular steps.

Variable		Healthy Controls			MS Patients			Interactive Effect of Variable	Effect of Group
		Mean (SD)		*P*-Value	Mean (SD)		*P*-Value	*P*-Value	*P*-Value
		Block 2	Block 6		Block 2	Block 6			
Explicit learning	Accuracy	66.36 (3.731)	67.85 (2.42)	0.309	68.25 (1.42)	68.33 (1.37)	0.838	0.358	0.457
	Speed	1.82 (0.57)	1.65 (0.53)	0.044	1.56 (0.286)	1.64 (0.384)	0.216	0.017	0.108
Implicit learning	Accuracy	68.33 (1.61)	68.25 (1.21)	0.878	86.67 (1.23)	69.00 (1.47)	0.517	0.572	0.230
	Speed	1.703 (0.543)	1.658 (0.454)	0.604	1.79 (0.49)	1.73 (0.48)	0.147	0.882	0.657

**Table 4:** Results of the paired *t*-test and the repeated measures ANOVA in regular steps.

Variable		Healthy Controls						MS Patients						Interactive effect of Variable	Effect of Group
		Mean (SD)					*P*-value	Mean (SD)					*P*-Value	*P*-Value	*P*-Value
		1	3	4	5	7		1	3	4	5	7			
Explicit Learning	Accuracy	66.92 (2.42)	67.67 (2.42)	67 (2.33)	67 (2.76)	68.67 (1.73)	0.01	66.17 (3.66)	66.25 (2.45)	67.92 (1.83)	68.42 (1.50)	68.25 (2.22)	0.09	0.13	0.845
	Speed	1.72 (0.57)	1.72 (0.53)	1.82 (0/57)	1.56 (0.62)	1.60 (0.5)	0.42	1.61 (0.40)	1.57 (0.31)	1.59 (0.31)	1.58 (0.32)	1.58 (0.36)	0.95	0.544	0.48
Implicit learning	Accuracy	66.92 (3.05)	68.5 (1.38)	68.08 (2.23)	69.33 (0.98)	69 (1.34)	0.008	66.25 (3.81)	67.43 (1.642)	68.5 (1.16)	68 (1.80)	69 (1.47)	0.012	0.369	0.38
	Speed	1.92 (0.66)	1.63 (0.68)	1.65 (0.63)	1.59 (0.52)	1.55 (0.50)	0.05	2.005 (0.64)	1.84 (0.70)	1.67 (0.37)	1.55 (0.56)	1.57 (0.60)	0.012	0.783	0.57
